# Emulsion-Coated Active Papers Extend the Storage Life of Tomato Fruit

**DOI:** 10.3390/foods14162774

**Published:** 2025-08-09

**Authors:** Laura Aguerri, Celia M. Cantín, Marinelly Quintero, Silvia Lóbez, Pedro Marco, Filomena Silva

**Affiliations:** 1 I3A—Aragón Institute for Engineering Research, University of Zaragoza, 50018 Zaragoza, Spain; 698694@unizar.es (L.A.); slobez@unizar.es (S.L.); 2 Estación Experimental Aula Dei (EEAD), CSIC, 50059 Zaragoza, Spain; cmcantin@eead.csic.es; 3 Centro de Investigación y Tecnología Agroalimentaria de Aragón (CITA), 50059 Zaragoza, Spain; pmarcomo@cita-aragon.es; 4 ARAID—Agencia Aragonesa para la Investigación y el Desarrollo, 50018 Zaragoza, Spain; 5 Faculty of Veterinary Medicine, University of Zaragoza, 50013 Zaragoza, Spain

**Keywords:** antifungal paper, fruit shelf life, EOs mixture, active emulsions, bio-based active material, natural antimicrobials

## Abstract

This study focused on developing emulsion-coated active papers with antifungal properties to extend the shelf life of tomatoes during home storage, thereby reducing food waste in households. First, a mixture of essential oils (EO_mix_)—composed of 33.3% oregano and 66.7% cinnamon leaf oils—was optimised through a mixture design and emulsified with cationic starches. Based on their stability and efficacy, two different emulsions containing 10% Tween 80, 6–8% EO_mix_, and HI-CAT or EVO cationic starches (82 or 84%, respectively) were selected and applied on paper. Then, the antifungal performance of the coated papers was tested in culture media against *Botrytis cinerea*, demonstrating strong antifungal activity in the vapour phase, effective for up to 31 days at 4 °C. In tests conducted with fresh tomatoes, the active papers improved fruit appearance and significantly reduced mould growth while maintaining overall sensory quality, indicating that these materials could extend tomato shelf life and thus offer a promising, low-cost, and biodegradable solution to reduce fruit waste at the consumer level, combining effective antifungal protection with good sensory performance in real-use conditions.

## 1. Introduction

Food waste is a global problem that urgently needs action. Besides the food that is produced and not consumed, other valuable resources are wasted in the process, such as water, soil, and land, and they come along with a carbon footprint and an increase in global greenhouse gas emissions [1,2]. Food loss and waste can occur at every stage of the food chain, from primary production to households. However, it is especially relevant in the latter. For instance, in 2022, from the 54.6 million tonnes of estimated food waste in the European Union, more than 30 million tonnes were wasted in households [3]. Among all food products, fruits and vegetables are highly perishable and suffer from rapid deterioration during both pre-harvest and post-harvest stages [4], accounting for almost half (45%) of all food losses and waste [5]. Given that the demand for agricultural products is expected to continue growing with the rise in global population [6], there is an urgent need to reduce their waste.

Fruit spoilage is often initiated by a wide range of microorganisms such as bacteria, yeasts or moulds [7,8]. Above all of them, fungal contamination is particularly important because moulds can survive in hostile environments [9], and most of them spread spores that can easily migrate from an infected plant or surrounding ambient to the fruit, where they can grow. In fact, moulds are considered the main cause of fruit waste at the post-harvest stage [10]. Besides, some of them are toxin producers (mycotoxins), which can pose a risk for animal and human health due to the broad range of adverse effects caused by these compounds [11,12,13]. The most common route of infection for pathogenic moulds is the natural holes or openings that exist in the fruits. In some fruits, the peduncular area can remain very fleshy and open, easily accessible to moulds, once the fruit is detached from the tree branch or bush. This is the case of tomato, in which most fungal infections occur after harvesting through the peduncle [14,15]. Some relevant fungal genera in fruits and vegetables are *Penicillium* spp., *Cladosporium* spp. and *Botrytis* spp. [16]. In tomato, postharvest decay induced by microorganisms such as *Rhizopus stolonifer*, *Alternaria alternata,* and *Botrytis cinerea* during postharvest handling, distribution and household level, is a serious problem worldwide [17,18,19]. The phytopathogenic fungus *Botrytis cinerea* (grey mould) causes serious pre- as well as postharvest diseases in many economically important crops, with a special focus on tomatoes, where mould is a devastating disease that causes serious financial losses worldwide [20,21]. *B. cinerea* infects tomatoes through injury resulting from harvesting or by direct penetration [22]. Its effect is more noticeable under favourable growth conditions such as low temperatures and high relative humidity, which corresponds to the typical post-harvest storage conditions for tomatoes [23].

To prevent fruit spoilage, including tomatoes, various approaches are being studied, with an emphasis on active packaging materials that incorporate compounds capable of extending food shelf life by interacting with the food itself or the surrounding atmosphere. Among the active compounds included in the material, natural antimicrobials such as essential oils (EOs) and plant-derived extracts are two of the most used, due to their low cost, biological origin and biodegradability [24,25,26,27]. Most EOs are Generally Recognised As Safe (G.R.A.S. by the U.S. Food and Drug Administration (FDA)) and commonly used as food flavouring agents [28]. They are composed of several volatile organic compounds that can exhibit antimicrobial or antioxidant properties, which make them ideal candidates to be used in a variety of ways to extend food shelf life [29,30]. Among all the EOs studied, it has been shown that cinnamon and oregano have a significant antimicrobial effect, especially against moulds [31]. For example, Manso et al. [32] tested the antifungal activity of different EOs (cinnamon bark, oregano, and clove) and their major constituents (cinnamaldehyde, carvacrol and eugenol), being cinnamon bark EO the most active agent against the tested strains of *Aspergillus flavus* and *Penicillium roqueforti*. In another study, Echegoyen & Nerín [33] developed a paraffin formulation containing cinnamon bark EO that was able to extend shelf-life of mushrooms.

Nevertheless, EOs have significant limitations that compromise their use in food packaging applications, including high volatility, poor water solubility, and potential sensory alterations at high concentrations [29,34,35,36,37,38]. To overcome these drawbacks, encapsulation techniques have been developed to protect EOs from environmental degradation and control their release over time [38,39]. For instance, modified starches are recently being explored as stabilisers and carriers in EO-based emulsions, enabling their incorporation into aqueous matrices and effectively dispersing EOs as fine droplets within the emulsion [40,41]. Furthermore, when applied as coatings, the starch matrix network increases the retention of EO droplets due to physical and chemical interactions, slowing their volatilisation and enabling sustained antifungal effect over time by means of gradual release [42].

In this study, two different active papers containing a mixture of essential oils (EOs) were developed to prevent fruit deterioration at the household level. To achieve a sustained release of antifungal volatile compounds from EOs, different starch-based emulsions were explored and incorporated into papers using a coating method. Furthermore, the EOs mixture (EO_mix_) used was optimised by means of a mixture design approach to minimise the quantity of active agent needed for antifungal effect and, therefore, their impact in the sensory properties of the food product. This combined approach—mixture design and the use of modified starches as carriers and emulsifiers—represents a promising step toward the development of sustainable; active materials; composed of cellulose; starch; and natural antimicrobials; with extended antifungal efficacy at minimum EO_mix_ concentrations. The performance of emulsion-coated active papers was evaluated through antifungal activity tests in culture media against *B. cinerea* in vapour phase, and on fresh produce, using tomato var. ‘Ibériko’, through the assessment of total fungal growth, fruit decay, and fruit quality during shelf-life. Furthermore, given the impact of EOs on food organoleptic properties, consumer’s perception was also evaluated through sensorial analysis of stored tomatoes with the final aim of finding a balance between antimicrobial effectiveness, fruit quality and consumer satisfaction.

## 2. Materials and Methods

### 2.1. Materials

Essential oils (EOs) were chosen after preliminary in-house results and acquired from different suppliers. Oregano EO (*Origanum vulgare*, OR) and Summer savory EO (*Satureja hortensis*, SH) were supplied by Matières Prèmieres Essentielles (Grasse, France). Cinnamon leaf EO (*Cinnamomum zeylanicum*, CL) and Red thyme EO (*Thymus zygis,* RT) were supplied by Esencias Martínez Lozano (Murcia, Spain).

For emulsion preparation, two cationic starches from Roquette Frères (Lestrem, France) were used: Stabilys EVO 280 (EVO) and Hi-Cat C373A (HI-CAT). To stabilise the emulsions, Tween^®^ 80 (synthesis grade; Scharlab, Spain) was used as a surfactant. The active material was developed using a cellulose paper provided by DORSAN Filtration (Barcelona, Spain) with 620 µm thickness and a grammage of 280 g/m^2^. This commercial paper complies with Recommendation XXXVI/1 of the German BfR on paper and board for food contact and is in accordance with Regulation (EC) 1935/2004 [43] on materials and objects intended to be in contact with food, according to the information provided by the supplier.

### 2.2. Methods

#### 2.2.1. Mixture Screening

To explore the possible added or synergistic effects of combining different EOs, a design of experiments software (DesignExpert v25.0) was used to minimise the amount of active agent needed to yield an antifungal effect. A mixture design was used using the percentage of the 4 EOs as the mixture components in an augmented simplex lattice design at a special cubic order, with a total of 30 experimental runs. The Minimal Inhibitory Concentration (MIC) in the vapour phase was established as the model response, with the purpose of minimising it.

#### 2.2.2. Pure Essential Oil Composition by GC-MS

Pure EO analysis was performed by direct liquid injection of 1 mL of each EO (oregano, summer savoury, cinnamon leaf, and red thyme) in split (1:800) mode using a CTC Analytics autosampler PAL3 system (Agilent Technologies, Barcelona, Spain) connected to a GC-MS system (GC 6890 N with an MS 5975) (Agilent Technologies, Spain). Chromatographic separation was carried out in an HP-5ms column (30 m × 0.25 mm × 0.25 μm) (Agilent Technologies, Barcelona, Spain). The initial column temperature was set at 40 °C, held for 2 min, and then raised to 220 °C at 10 °C/min and held for 3 min. Helium (99.999%, Messer Iberica, Barcelona, Spain) was used as carrier gas at a flow rate of 1.0 mL/min. MSD source was set at 250 °C, with the detector being operated in Scan mode in the range 40–400 *m*/*z*. The obtained chromatograms were analysed using OpenLab ChemStation (Agilent Technologies, Barcelona, Spain), and compounds were identified with the NIST Chemistry WebBook library, using Match and Retention Index parameters. The bioactive components composition (%) of each EO can be found in the Supplementary Information (Oregano EO—Supplementary Table S1; Summer savoury EO—Table S2; Cinnamon leaf EO—Table S3; Red thyme EO—Table S4).

#### 2.2.3. Antifungal Activity Tests

##### Fungal Strain

Antifungal activity tests were performed using the fungal strain *Botryotinia fuckeliana* CECT 2100 (teleomorph of *Botrytis cinerea*) obtained from the Spanish Culture Type Collection (CECT; Valencia, Spain). Mould spores were suspended in water supplemented with 0.1% (*v*/*v*) Tween^®^ and 20% (*v*/*v*) glycerol and stored at −80 °C. Prior to each experiment, *B. fuckeliana* was grown for 12 days at 25 °C in Sabouraud Dextrose Agar (SDA) (Scharlau, Spain) plates.

For antifungal activity tests in culture media, inoculum preparation was carried out following EUCAST guidelines on spore/conidia suspension preparation [44]. Briefly, fungal isolates were subcultured on SDA for 12 days to achieve proper sporulation. Then, sterile water supplemented with 0.1% Tween^®^ 20 was used to cover the colonies. Conidia were carefully rubbed with a sterile cotton swab and transferred to a sterile tube. The suspension was homogenised using a vortex, and the concentration was adjusted to 10^6^ CFU/mL with sterile distilled water.

##### Pure EOs

**Figure 1:**
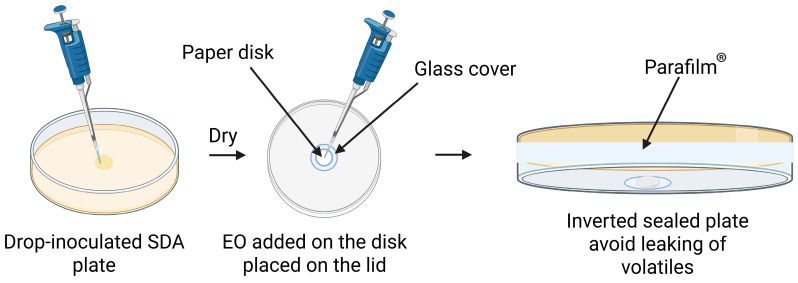
Schematic representation of the vapour phase contact assay (inverted method) used for the determination of Minimal Inhibitory Concentrations (MIC). Fungal-inoculated plates were exposed to different volumes of essential oils (EOs), applied onto a paper disk placed on the lid of the plate, avoiding direct contact with the mould. The plates were then Parafilm-sealed to ensure that volatile compounds remained inside.

Antifungal susceptibility testing of the volatile fraction of each EO was carried out in indirect contact (vapour phase) by using an inverted Petri dish method (Figure 1). The procedure followed was adapted from Tullio et al. [45], with slight modifications. SDA plates were inoculated in the centre of the plate with 10 µL of a mould spore suspension at a concentration of 10^6^ CFU/mL and allowed to dry in a flow safety cabinet. Meanwhile, a paper disk was placed in the centre of the inner part of the plate lid previously covered with a glass cover. Once the spore suspension drop was dry, the appropriate volumes of EO were placed in the paper disk at the lid, and the plate was immediately inverted and Parafilm^®^ sealed, with no contact between the disk and the agar. Parafilm^®^ sealing prevented the leakage of volatile compounds. For each plate, a single paper disk was used. EO volumes tested ranged from 0.625 µL to 10 µL (serial twofold concentrations). The air volume available inside of the sealed SDA plates was calculated to be 38 cm^3^ (90 mm Petri dish filled with 20 mL SDA). Plates were incubated at 25 °C for 7 days. Minimal Inhibitory Concentration (MIC) was defined as the minimum concentration of EO that resulted in no visual fungal growth (total inhibition) after a 7-day incubation period (Supplementary Figure S1) and expressed in terms of μL EO added to the plate per litre of available air inside the sealed plate. MIC values indicate fungal susceptibility to an antimicrobial compound (EO or EO_mix_), with lower MIC values signifying higher sensitivity of the fungal strain to the EO or EO_mix_ and higher MIC values indicating increased resistance. Each experiment was repeated at least three times, and the modal MIC values were selected.

##### Mixture of EOs

For each antifungal activity assay, the EO mixture (EO_mix_) was freshly prepared according to the mixture design proposal. A total volume of 100 µL for each mixture was prepared and homogenised prior to use. The set of 30 experimental runs was conducted in the vapour phase in the same experimental conditions as the vapour assays of the pure EOs (Section Pure EOs).

#### 2.2.4. Emulsion Preparation

Several emulsions were prepared by testing different cationic starches, EO_mix_ concentrations (2–10% *v*/*v*), and the presence or absence of surfactant (Tween^®^ 80 at 10% *v*/*v*). A summary of the emulsions tested in the preliminary assays is shown in Table 1. All these emulsions were coated onto the paper as described in Section 2.2.5 and evaluated for their vapour-phase antifungal activity against *B. cinerea* over 31 days of cold storage (4 ± 2 °C) as described in Section 2.2.6. According to the preliminary results obtained, two EO_mix_-emulsions (A and B) were selected due to their stability and preliminary antifungal activity tests. EO_mix_ emulsion A consisted of 82% HI-CAT starch solution, 10% Tween^®^ 80, and 8% EO_mix_, while EO_mix_ emulsion B was prepared with 84% EVO starch, 10% surfactant, and 6% EO_mix_. Percentages are expressed in *v*/*v*. These emulsions were chosen to ensure maximum antifungal activity while minimising the risk of altering the organoleptic properties of fresh tomatoes in the experiments.

Briefly, EO_mix_-emulsions (10 mL) were prepared by mixing the compounds using a mechanical stirrer (IKA-Werke; Staufen, Germany) with an R 3004 blade stirrer. First, the surfactant (1 mL) was added to the cationic starch solution, and subsequently, the EO_mix_ (0.6 or 0.8 mL for EVO or HI-CAT, respectively) was added and homogenised at 1000 rpm for 10 min at room temperature. Prior to their use, starches were dissolved at a low concentration in distilled water at 95 °C with magnetic stirring for 30 min to allow gelatinisation, according to the manufacturer’s instructions. EVO starch solution was prepared at a concentration of 10% (*w*/*v*), while HI-CAT starch solution was prepared at 8% (*w*/*v*), due to its higher density.

#### 2.2.5. Paper Coating and Storage

Filter paper samples with a 620 µm thickness and a grammage of 280 g/m^2^ (20 cm × 10 cm) (Dorsan Filtration, Spain) were coated with 3 mL of the emulsion using a coater machine (K Control Coater, RK Printing Instruments Ltd.; Royston, UK). After the coating, the papers were allowed to dry at room temperature.

A set of six replicates of each emulsion-coated paper (EO_mix_-emulsion A-coated papers (active papers A) and EO_mix_-emulsion B-coated papers (active papers B)) were prepared. Immediately after drying, the papers were stored in zip-lock bags and kept in the fridge at 4 ± 2 °C. Additionally, control papers (A and B) were prepared without the EO_mix_ but including the starch and surfactant at the same concentrations used in each emulsion.

#### 2.2.6. Assessment of Active Paper Stability

To assess the stability of the active paper, the antifungal activity in culture media in the vapour phase against B. cinerea and the volatile profile through time of the developed papers were studied. The antifungal activity of both active papers stored at 4 ± 2 °C was evaluated over time. Antifungal activity assays were conducted at 3, 10, 17, 24, and 31 days after coating. At each time point, the vapour-phase antifungal activity against *B. cinerea* was assessed using paper samples of 1 cm^2^ (3 independent replicates for active and control paper samples) as previously described. Fungal growth was determined after a 7-day incubation period.

At the same time intervals, the amount of EO_mix_ compounds present in the stored paper was evaluated by Headspace-GC/MS as follows. HS analysis was performed in the headspace environment of 20 mL glass vials using a 2.5 mL gas-tight syringe according to the manufacturer’s instructions (Agilent Technologies, Barcelona, Spain). Headspace was sampled (1 mL) after extraction of volatile compounds from 0.2 g of the active papers (A and B) at 60 °C for 15 min with agitation. The HS syringe case was maintained at 105 °C to avoid any condensation of volatiles in the sampled headspace volume before injection. Inlet temperature was set at 250 °C. Volatile extraction and headspace sampling procedures were performed automatically using a CTC Analytics autosampler PAL3 system from Agilent Technologies (Barcelona, Spain) connected to a gas chromatograph system (GC 6890 N with an MS 5975) (Agilent Technologies, Barcelona, Spain). Chromatographic separation was carried out in an HP-5ms column (30 m × 0.25 mm × 0.25 μm) (Agilent Technologies, Spain). All samples were injected in splitless mode. The initial column temperature was set at 50 °C, held for 5 min, and then raised to 100 °C at 5 °C/min, followed by an increase to 300 °C at 10 °C/min and held for 2 min. Helium (99.999%, Messer Iberica, Spain) was used as carrier gas at a flow rate of 1.2 mL/min. MSD source was set at 250 °C, with the detector being operated in Scan mode in the range 45–450 *m*/*z*. The obtained chromatograms were analysed using OpenLab ChemStation (Agilent Technologies, Spain) and compounds were identified with the NIST Chemistry WebBook library, using Match and Retention Index parameters. This analysis was performed by triplicate on three independent active paper samples.

#### 2.2.7. Evaluation of Active Papers on Fresh Tomatoes

For the assay on fresh produce, freshly harvested tomato fruit (*Solanum lycopersicum*, cultivar ‘Ibériko’) (n = 500) from Murcia (Spain) at the mature red stage were used. The tomatoes arrived at the laboratory the day after being harvested and were selected and sorted based on their uniformity in size, absence of defects and homogeneous maturity (measured with DAmeter^®^, Turony, Italy). Subsequently, the selected fruits (n = 486) were packaged in batches of six tomatoes in 81 plastic containers with lids (21 cm × 28 cm × 14 cm), simulating the fruit and vegetable drawer of the refrigerator. Containers were divided into three even batches: One-third were control containers (without paper), another third contained one sample of paper A, and the rest contained one sample of paper B. The paper sample (20 cm × 10 cm) was taped to the lid to prevent contact between the tomatoes and the paper.

After an initial basic quality evaluation, both control and treatment containers were cold-stored at 4 ± 2 °C for 7, 14, 21, and 28 days, with tomatoes being evaluated at each storage time point (D7, D14, D21, and D28) and again after an additional 2 days of shelf life at 20 °C following each storage period (simulating kitchen counter short storage; D7+2, D14+2, D21+2, and D28+2). Three repetitions were used for each treatment and storage time combination.

##### Initial Fruit Quality

Fruit quality was initially evaluated in 15 fruits (Supplementary Table S5). Fruit skin colour was determined using a HunterLab colour difference chromameter machine (Minolta, Tokyo, Japan) in two opposite cheeks of each tomato. A white and black ceramic plate was used to standardise the instrument every time for accuracy. The reflected L*, a*, and b* colour values and the hue angle (h) were recorded. Fruit firmness was evaluated by using a non-destructive mechanical sensor (Durofel, CTIFL, Paris, France), which measures the superficial retraction under the strain of a force and is expressed in dimensionless units. Total soluble solids (TSS) were measured in three repetitions of tomato juice by using a digital refractometer (Model: PR-32 α, ATAGO Co., Ltd., Tokyo, Japan). Three tomato juices were extracted by homogenising an edge of five tomatoes using a food blender and then filtered using a muslin cloth. One to two drops of clear juice were placed on the prism. Between each sample, the prism was washed with distilled water. The resulting values were expressed as ° Brix. The titratable acidity (TA) of the tomato was determined by titrating 3 repetitions of 5 mL tomato juice against 0.1 N NaOH. Titratable acidity was expressed as a percentage of citric acid.

##### Decay Incidence

The incidence of fungal rots was measured following the method proposed by Badawy and Rabea [46], with some modifications, and determined by Equation (1):

Decay incidence (%) = (number of infected tomatoes/total number of tomatoes) × 100(1)

##### Decay Index

The decay index (DI) was evaluated visually according to the methodology proposed by Perdones et al. [47] following a visual scale (Supplementary Figure S2). The results of fungal presence, mechanical damage, and physical skin deterioration were calculated using the following Equation (2):

Decay index (DI) = (1n + 2n + 3n + 4n)/N(2)

where n = number of fruits classified at each level of the damage scale and N = number of total fruits analysed in each treatment per evaluation point.

##### Microbial Growth

The biocidal activity of antifungal papers on the growth of moulds, yeasts, total mesophilic anaerobes, bacterial species, and enterobacteria was evaluated. Samples were analysed according to ISO Standard 6887-1:2017 [48] adapted to this product. To do so, at each evaluation point, one fruit from each repetition was placed in a sterile bag with the same weight of the fruit in mL of sterile saline peptone water (0.1% peptone + 0.85% NaCl) and was carefully shaken and massaged for 5 min to drag the entire load of microorganisms existing on the surface, avoiding damage and breakage of the fruit. Each sample was serially diluted, and aliquots (0.1 mL) of the microbial dilutions were surface plated. Culture media and incubation conditions for the enumeration of each microbial group were plate count agar (PCA) (Merck) during 72 h at 30 ± 1 °C for total aerobic mesophilic microorganisms [49]; violet red bile glucose (VRBG) (Oxoid) during 24 h at 30 ± 1 °C for the *Enterobacteriaceae* family [50]; and dichloran rose-bengal chloramphenicol agar (DRBC) (Merck) during 4 days at 25 ± 1 °C for moulds and yeasts [51]. The value presented for the microbial count on each sampling day, expressed as log CFU·g^−1^, was the average of three samples per packaging condition.

##### Consumer Acceptance

For consumer evaluation, tomato samples were evaluated after 7, 14, and 21 days of cold storage at 4 ± 2 °C followed by 2 days of shelf life at 20 °C (simulating short-term kitchen counter storage in households). Analyses were conducted according to ISO 11035:1994 [52] standards. Samples of control fruits and those stored with the antifungal papers were placed on white plates and immediately presented to a panel of at least 30 consumers. The tomatoes were cut longitudinally into 8 edges with skin, always of the same width (approx. 1.5 cm). The consumers were volunteers from the staff working at the Aula Dei Campus (Zaragoza). Each sample was identified by a three-digit random code, and the order of presentation of the piece was randomised for each taster. Mineral water was used as a palate cleanser between tastings. Each consumer evaluated all samples and was asked to indicate their degree of liking/disliking and external appearance using a 9-point hedonic scale (1 = I do not like it at all to 9 = I like it extremely). Firmness, juiciness, mealiness, tomato aroma, and off-flavours were also evaluated with a 9-point hedonic scale (1 = no perception to 9 = very strong perception of each attribute). The study was conducted in accordance with the Declaration of Helsinki, and the protocol was approved by the Institutional Review Board of the Estación Experimental de Aula Dei (EEAD-CSIC) on 14 February 2023.

#### 2.2.8. Statistics

Statistical analysis of the results was performed using IBM SPSS software (version 29.0.0.0). Differences in mean scorings were analysed with a one-way ANOVA and separated using Tukey’s significant difference test (*p* < 0.05). Angular transformation was performed on data in percentage before proceeding with ANOVA to equalise variances of proportions [53].

## 3. Results and Discussion

### 3.1. Antifungal Activity of Pure EOs

Results of antifungal activity in the vapour phase of each EO are listed in Table 2.

The four EOs showed high antifungal activity, with total mould inhibition being obtained with added volumes of 1.25 to 2.50 µL of the different EOs. OR and CL showed the highest antifungal activities, with MIC values of 32.9 μL EO/L air. These results can be due to the highest percentage of carvacrol (Supplementary Table S1) and eugenol (Supplementary Table S3) presented by these EOs, respectively, accounting for more than 50% of the total composition of the EO. Several studies have shown that carvacrol is more effective against moulds than other terpenoid phenols such as thymol, eugenol, or menthol [54,55]. However, when these compounds are included in a mixture, such as an EO, such differences get slightly diluted, and similar results might be obtained for EOs containing carvacrol or eugenol as major components [54], probably due to the additive interactions or synergistic effects of these compounds with the other EO components. The results obtained correlate with those found by Xiang et al. [56], where oregano EOs exhibited one of the highest antifungal activities against *Aspergillus flavus* in the vapour phase. Similarly, Abdolahi et al. [57] tested the antifungal activity of *Summer savory* EO (SH) against *B. cinerea* in the vapour phase and reported a total inhibition of the mould when adding 5 μL of SH, while in our case, only 2.5 μL of SH (MIC value at 65.8 μL EO/L air) were needed to inhibit completely *B. cinerea* growth.

Even so, minor differences are commonly found in the literature when comparing EO MICs in the vapour phase. For instance, Álvarez-García et al. [58] studied the antifungal activity of samples of *Origanum vulgare* and *Thymus vulgaris* EOs coming from different suppliers against *B. cinerea* and found that in the case of *O. vulgare*, both MICs were the same (22.73 µL EO/L air), but they were slightly different in the case of *T. vulgaris* (MIC at 45.45 and 22.73 µL EO/L air). This finding evidenced that, even if the EO comes from the same plant species, some differences in their antifungal properties can be found. In that regard, our results are consistent with the ones found in literature, although there is a lack of standardisation in terms of culture media, incubation times, and inoculum concentration between studies when performing these vapour phase assays, rendering results comparison difficult.

### 3.2. Screening of Mixtures and Optimal Combination

The 30-mixture compositions and MIC results are shown in Table 3. As shown, MIC values ranged from 16.4 to 65.8 μL EO_mix_/L air (corresponding to an added volume of 0.625 μL to 2.50 μL EO_mix_). Among all the combinations tested, only one yielded a MIC result lower than the MICs of each EO component alone (assay 18: 0.33% OR + 0.67% CL, with a MIC value of 16.4 μL EO_mix_/L air), this being the selected combination to be incorporated in the emulsions.

When looking at the binary mixtures, it is shown that the combination of two EOs with the same activity at the pure state yielded different behaviours when combined. Thus, in the case of the combinations of SH + RT (assays 6 and 29), their antifungal activity was the same as when separated. However, in the case of the combinations of the most active EOs (CL + OR; assays 7 and 18), this was not the case. When CL was at a higher rate in the mixture (assay 18), the antifungal activity was higher than the EOs alone; while in the case of CL being at a lower proportion in the mixture (assay 7), the antifungal activity was similar to the pure EO (MIC at 32.9 μL EO/L air). In the case of CL, this effect of lowering the MIC when CL is present at a higher percentage in the mixture was also observed when it was combined with SH or RT, showing a lower MIC value (32.9 μL EO_mix_/L air in assays 15 and 23) than the MIC of SH or RT alone (65.8 μL EO/L air). The presence of OR in any of the combinations with SH (assays 12 and 30) or RT (assays 13 and 17) enhanced the antifungal activity when compared to the effect of SH or RT alone (MIC 32.9 μL EO_mix_/L air versus 65.8 μL EO/L air).

In the case of ternary mixtures, the presence of OR at any rate in the mixture ensured the MIC at 32.9 μL EO_mix_/L air (assays 3, 16, 20, and 26). The only assay in which the OR was not present (assay 4) showed a lower antifungal activity (MIC at 65.8 μL EO_mix_/L air). This finding, combined with the results on the binary mixtures, suggests that OR has a significant influence on lowering the MIC value of the mixtures.

When the four EOs were combined, a higher proportion of CL in the mixture lowered the antifungal activity (assay 1, MIC at 65.8 μL EO_mix_/L air). This result contradicts what was seen in the binary mixtures and suggests that some antagonistic effect could be found between the EOs. In the rest of the quaternary mixtures (assays 10, 19, and 22), the MIC value was found to be 32.9 μL EO_mix_/L air.

In general, these results are in line with the ones presented in the literature, where synergistic or additive effects between EOs due to their different antimicrobial mechanisms of action have been reported several times [59]. By combining different EOs and, therefore, different pathways of antimicrobial action in microorganisms, a decrease in the minimal inhibitory concentration can be obtained. This effect could allow reducing the EO concentration in food packaging thus reducing the negative effects on organoleptic attributes while maintaining antimicrobial efficacy. For example, the synergistic antifungal effect of a combination of mustard EO and cinnamon bark EO was observed by Clemente et al. [60] against the bread mould *Rhizopus stolonifer*. At the cinnamon:mustard EO (100:8) combination, the mustard EO provided high antifungal activity, while the cinnamon bark EO masked the unpleasant mustard odour. Besides, it was effectively used for preventing mould spoilage in Spanish bread. Nikkhah et al. [61] evaluated the antifungal activity of thyme, cinnamon, marjoram, and rosemary EOs and their combinations. In their study, pure thyme and cinnamon EOs were the most active EOs and synergistic effects against *B. cinerea* were found in binary combinations of cinnamon and thyme EO, and in ternary combinations of cinnamon, thyme and marjoram EOs. Moreover, carvacrol and thymol, which are the main terpenoid phenols present in the essential oils of oregano and thyme, respectively, have shown in vitro additive antimicrobial effect against Gram-positive and Gram-negative bacteria and fungi [62,63].

### 3.3. Paper Stability Through Time (Volatile Composition and Culture-Media Assays)

**Figure 2:**
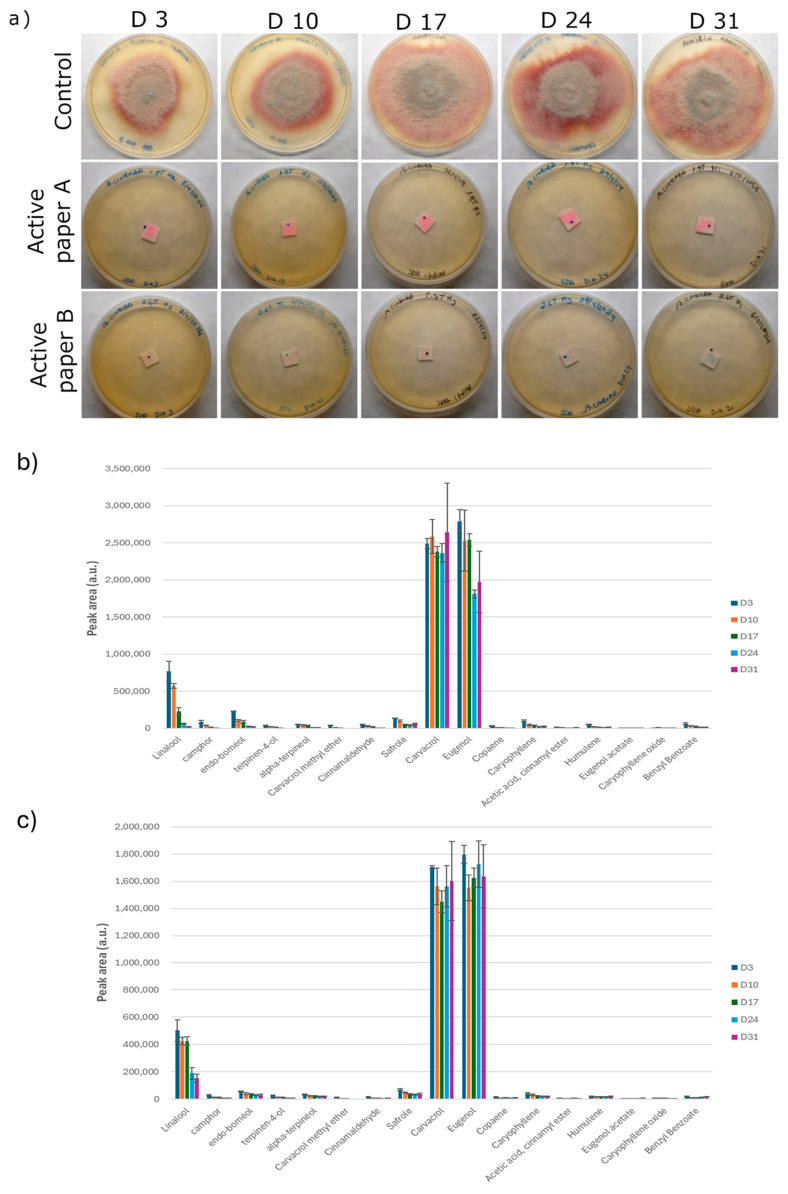
Antifungal activity against *B. cinerea* (**a**) and volatile composition analysis by HS-GC/MS of active papers A (**b**) and B (**c**) along storage at 4 ± 2 °C (3, 10, 17, 24, and 31 days after paper coating). Volatile compounds are presented as mean area ± SD of each individual peak.

The results on active-paper stability at 4 ± 2 °C over time are shown in Figure 2. When stored at 4 ± 2 °C, both active papers exhibited high antifungal activity against *Botrytis cinerea,* with no mould growth observed throughout the entire 31-day storage period (Figure 2a). As the papers are intended for use in post-harvest cold storage, 31 days at 4 ± 2 °C proved enough to maintain paper activity in both cases. Volatile analysis on both active papers revealed that the main components from both OR and CL EOs can be found by HS-GC/MS analysis (Figure 2b,c), such as camphor, endo-borneol, carvacrol, carvacrol methyl ether from oregano EO; cinnamaldehyde, safrole, copaene, cinnamyl ester acetate, eugenol acetate, benzyl benzoate, and most of the eugenol from cinnamon leaf EO. Compounds such as linalool, terpinen-4-ol, alpha-terpineol, caryophyllene, humulene, and caryophyllene oxide can be found in both EOs (Supplementary Tables S1 and S3). As expected, active paper B showed lower levels of EO compounds due to its lower EO_mix_ amount in the emulsion (6%) in comparison with active paper A, which has been coated with an 8% EO_mix_ emulsion. The main components found in both active papers were carvacrol and eugenol, accounting for 75–80% of the total compounds identified in each active paper. Additionally, the results showed that the levels of carvacrol and eugenol in both active papers did not significantly decrease during cold storage (*p* < 0.05), except for the eugenol levels in paper A, which showed a significant decrease at 24 and 31 days of storage. On the other hand, other active compounds, such as linalool, also present in both papers, significantly decreased over time. These results suggest that the antifungal activity of the active papers could be mainly attributed to carvacrol and eugenol, as their consistent levels over the 31 days of cold storage were correlated with sustained antifungal activity.

### 3.4. Evaluation of the Active Packaging on Tomatoes

#### 3.4.1. Decay Incidence

**Figure 3:**
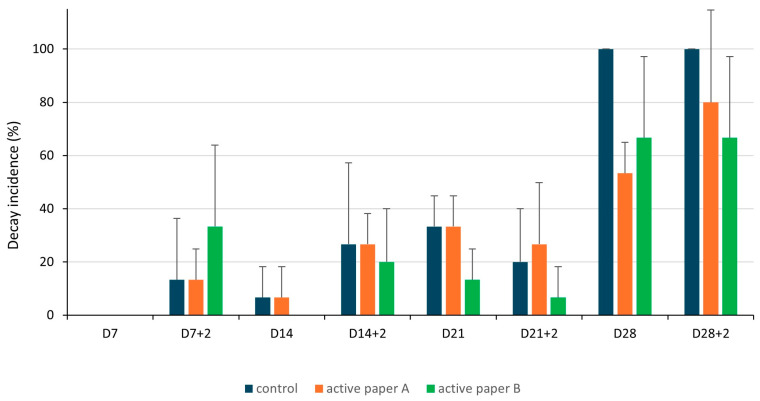
Decay incidence (%) of tomatoes after 7, 14, 21, and 28 days of cold storage (4 ± 2 °C) (D7, D14, D21, and D28) and after the cold storage followed by 2 days of shelf life at 20 °C (D7+2, D14+2, D21+2, and D28+2), under control, active paper A and active paper B treatments. Different letters for each evaluation time indicate significant differences among treatments according to Tukey’s test at *p* < 0.05.

As shown in Figure 3, after 7 days of cold storage plus 2 days of shelf life at 20 °C, some degree of decay was already observed in the control and both treatments (active paper A and B). No significant differences in decay incidence were observed between the control and both active papers after 7, 14, and 21 days of cold storage, with or without an additional 2 days of shelf life at 20 °C. However, after 28 days of cold storage, control tomatoes showed a significantly higher decay incidence compared to those stored with paper A. Specifically, on the last day of cold storage (28 days), 100% of the control fruits showed rot, while the fruits stored with papers A and B exhibited rot in only 53.33% and 66.67% of the fruits, respectively. This effect was also observed when the fruits were transferred to 20 °C for an additional 2 days of shelf life (100%, 80.00%, and 66.67% rot in the control, paper A, and paper B, respectively). At this point, tomato decay in the control fruit was significantly higher than in tomatoes under paper B, although it was not statistically different from paper A due to the high variability among samples within the same treatment. This high variability is reflected in the standard deviation values and likely results from biological differences in fruit susceptibility and uneven disease progression under severe infection pressure. Previous works have reported better protection against pathogen attack on fruit with higher concentrations of essential oils in the formulations [64,65]. However, in our study, no statistically significant differences were observed among the papers, despite active paper A having a higher EOs concentration than active paper B (8% vs. 6%, respectively).

#### 3.4.2. Decay Index

As expected, the decay index (Table 4) increased as the storage period progressed. Even under cold storage at 4 ± 2 °C, signs of damage were observed immediately after 28 days of cold storage. However, significant differences between the control and the treatments became evident after 28 days of cold storage at 4 ± 2 °C, followed by two days of shelf life at 20 °C. The control fruits exhibited a decay index of 2.5, while the fruits stored with papers A and B had decay index values of 1.7 and 1.5, respectively, indicating a lower incidence of damage due to infection compared to the control. No differences were observed in the decay index between the two active papers.

These results coincide with those obtained by other authors such as Buendía-Moreno et al. [66], who evaluated the effect of a coating that included a mixture of essential oils (carvacrol, oregano, and cinnamon) on the shelf life of fresh tomatoes and reported a reduction in the decay rate of fruits by up to 15% after a cold storage period of 6 days at 8 °C followed by 12 days of shelf life at 25 °C.

#### 3.4.3. Microbial Growth

**Figure 4:**
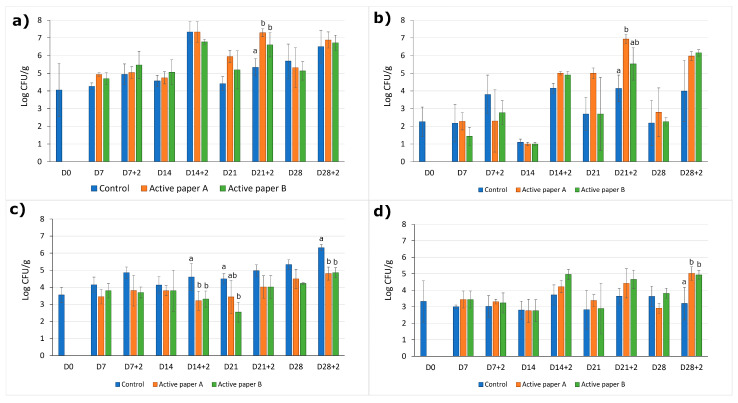
Evolution of (**a**) mesophilic aerobic microorganisms, (**b**) *Enterobacteriaceae* family, (**c**) molds, and (**d**) yeasts on the surface of tomatoes under control, active paper A, and active paper B treatments after 7, 14, 21, and 28 days of cold storage at 4 ± 2 °C (D7, D14, D21, D28), and after 7, 14, 21, and 28 days of cold storage (4 ± 2 °C) plus 2 days of shelf life at 20 °C (D7+2, D14+2, D21+2, D28+2). Different letters indicate significant differences between treatments each day according to Tukey’s test (*p* < 0.05).

Results on the performance of active papers on microbial growth in tomatoes are shown in Figure 4. The evolution of mesophilic aerobic microorganisms, *Enterobacteriaceae* family, moulds, and yeasts in tomatoes treated with active paper A, active paper B, and control samples was monitored over 28 days at 4 ± 2 °C, with additional observations after 2 days at room temperature (20 °C).

In the case of mesophilic aerobic microorganisms and those from the *Enterobacteriaceae* family (Figure 4a,b), active papers did not exhibit high antimicrobial efficacy, especially after room temperature exposure, when they showed the highest counts of these groups. Particularly, mesophilic aerobic microorganisms exhibited the highest counts during all assays. In control samples, counts increased from 4.1 log CFU/g on day 0 to 5.7 log CFU/g by day 28, increasing to 6.5 log CFU/g after exposure during 2 days at room temperature. Active paper treatments did not significantly suppress these counts, even showing higher counts than the control after 21 days of cold storage plus 2 days of shelf life. In the case of *Enterobacteriaceae* family, counts remained low in control samples during refrigerated storage (≈2.2 log CFU/g), increasing to 4.0 log CFU/g after exposure to room temperature. In contrast, active paper-treated samples showed significant increases under warmer conditions (>6.2 log CFU/g). Statistical analysis confirmed these differences after 21 days of cold storage plus an additional 2 days of exposure to room temperature, where counts on tomatoes with active paper A were statistically higher than on control tomatoes. No significant differences were observed on other days.

When looking at the mould growth counts (Figure 4c), both active papers reduced mold growth compared to those tomatoes stored without paper (control) during refrigerated storage and after exposure to room temperature, showing significantly better results than control tomatoes after 21 days of cold storage and after 14 and 28 days of cold storage plus an additional 2 days of shelf life. At those points, both papers showed comparable inhibition effects, although active paper B exhibited slightly better inhibition than paper A after 21 days of cold storage. Mould counts in the control samples increased by 1.8 log CFU/g over 28 days of cold storage, escalating by 0.8 log CFU/g after exposure to room temperature (20 °C). Active papers effectively reduced mould growth during both refrigerated storage and after room temperature exposure. Counts in Active paper A and B samples did not exceed 4.9 log CFU/g, with active paper B maintaining the lowest mould counts throughout the study.

Yeast populations in control samples (Figure 4d) remained relatively stable during all times of storage. Tomatoes stored with active papers showed a higher number of yeasts after room temperature exposure from day 14 (D14+2, D21+2, and D28+2), although this increase was only significant on the last day of the assay (D28+2), when tomatoes stored with paper showed a higher content of yeasts compared to the control tomatoes.

Overall, these results indicate that active papers were effective in suppressing mould growth, even after exposure to room temperature, suggesting that the emulsion successfully protected the components of the EO_mix_ while enabling their sustained release from the starch matrix on the coated surface of the papers, even at higher temperatures. However, the developed active papers had limited efficacy against mesophilic aerobic microorganisms, the *Enterobacteriaceae* family, and yeasts under warmer conditions (after the room temperature exposure). These results correlate with other studies, such as Buendía-Moreno et al. [67], who developed an active cardboard box for tomato storage containing EOs encapsulated into cyclodextrins. In their study, their active box significantly reduced mould growth on tomatoes but had limited effect on mesophiles, *Enterobacteriaceae* and other bacterial groups. It is important to note that tomato decay is mainly caused by mould microorganisms during postharvest. Therefore, the observed lower decay incidence and decay index in tomatoes treated with active papers A and B, particularly after 28 days of cold storage, can be correlated with the microbial results, which demonstrated a significant reduction in mould growth in these treatments. This suggests that the antifungal properties of the active papers, effective in suppressing mould, play a crucial role in reducing overall decay caused by microbial infection, especially during extended storage periods.

#### 3.4.4. Consumer Perception

Finally, a sensory evaluation was also carried out through a consumer panel to evaluate the effect of the two active papers on the organoleptic attributes of the tomato (Table 5).

No significant differences were found between control tomatoes and tomatoes stored with active papers regarding parameters such as appearance, firmness, juiciness, mealiness, and tomato flavour at any storage time. However, some panellists reported the appearance of strange flavours (off-flavours) in the tomato samples stored with active papers. Indeed, after 7 days of cold storage followed by 2 days of shelf life, significant differences were described by consumers in the intensity of off-flavours in the fruit stored with active papers. However, no differences in the global tomato acceptance from the three lots evaluated were observed.

Surprisingly, after 14 days of cold storage followed by 2 days of shelf life, a significantly greater intensity of off-flavors was reported for the fruits stored with the active paper with the lower EOs concentration (active paper B), which obtained a score of 4.54, while the control fruits and the fruits stored with paper A showed lower scores (2.88 and 3.51, respectively).

After 21 days of cold storage followed by 2 days of shelf life, the presence of both papers gave rise to a greater intensity of off-flavours in the fruits. As in the previous evaluations, these differences did not produce significant differences in the global acceptance of the tomato. These results indicate that, although the antifungal papers induced an alteration of the tomato flavour, this effect was not negative for the consumer, obtaining global acceptance values similar to those of the control fruits. Campos et al. [68] showed similar results when evaluating thermoplastic starch films containing thymol and carvacrol EOs in strawberries.

As explained before, flavour notes are one of the main disadvantages of EOs when exploring their antimicrobial activity in food-related applications [68,69,70]. Thus, a sensory evaluation should be included when evaluating an active material with EOs. For example, Chaidech & Matan [71] developed a reusable paper box containing cardamom EO for storing rambutan fruit and preventing the growth of *Pestalotiopsis* spp., resulting in the extension of rambutan’s life up to 7 days more in comparison to control boxes without EO. However, no sensory evaluation was described, and it may well be an obstacle for industry development. In our case, consumers’ opinions have been explored, yielding an overall acceptance of the active papers’ performance. Even so, more research is needed to diminish the flavour notes described by some panellists.

## 4. Conclusions

Among the EOs tested, OR and CL displayed excellent antifungal properties against *B. cinerea* in the vapour phase. Additionally, mixing CL and OR at a specific rate using a mixture design resulted in additive or synergistic antifungal effects, making this approach ideal for minimising the amount of active agent needed while maintaining its fungal inhibition. Emulsions proved to be a good alternative for incorporating the EO_mix_ into paper, as the antifungal properties of the active papers were preserved during cold storage, making them effective for food storage purposes. In assays carried out on fresh tomatoes, the developed active papers enhanced tomato appearance and extended their storage and shelf life by reducing mould growth and fruit decay. These results suggest that these active papers could potentially be effective for other fruits and vegetables typically affected by *B. cinerea* or similar moulds. Furthermore, the overall sensory acceptance of tomatoes stored with the active papers, combined with their ease of use, indicates that these active materials could be easily used throughout the food supply chain from post-harvest to retail. However, further studies are needed to minimise the impact of the active papers on tomato flavour. Additionally, the industrial development of these active papers is feasible due to the low cost of materials and the simple, fast coating already employed in the paper manufacturing industry. Finally, the fact that the developed active papers are biobased, composed of starch, cellulose, and EOs, and are aimed at reducing food waste makes them a properly thought-through strategy in terms of sustainability that can easily reach our households.

## Figures and Tables

**Table 1:** Composition (% *v*/*v*) of starch solution, surfactant, and EO_mix_ in emulsions tested.

**Cationic starch**	EVO or HI-CAT
**Starch solution**	80–98%
**Tween 80**	0% or 10%
**EO_mix_**	2–10%

**Table 2:** Minimal Inhibitory Concentrations (MICs) values and corresponding added volumes of pure essential oils (EOs). MICs are expressed as volume (μL) of pure EO added per volume (L) of air available inside the plate.

EO	SH	OR	CL	RT
**MIC** **(µL EO/L air)**	65.8	32.9	32.9	65.8
**Added volume (µL)**	2.50	1.25	1.25	2.50

SH = Summer savoury EO; OR = Oregano EO; CL = Cinnamon leaf EO; and RT = Red thyme EO.

**Table 3:** Results of mixture screening: Minimal inhibitory concentration (MIC) and corresponding mixture volumes added. MICs are expressed as volume (μL) of mixture of essential oil (EO) added per litre of air available inside the plate. For each assay, the proportions (expressed on a per-unit basis) of each added EO are listed.

Assay	SH (%)	OR (%)	CL (%)	RT (%)	MIC (µL EO/L air)	Added Volume of Mixture (µL)
**1**	0.125	0.125	0.625	0.125	65.8	2.50
**2**	-	-	-	1	65.8	2.50
**3**		0.333	0.333	0.333	32.9	1.25
**4**	0.333	-	0.333	0.333	65.8	2.50
**5**	-	-	0.333	0.667	65.8	2.50
**6**	0.333	-	-	0.667	65.8	2.50
**7**	-	0.667	0.333	-	32.9	1.25
**8**	1	-	-	-	65.8	2.50
**9**	-	1	-	-	32.9	1.25
**10**	0.125	0.625	0.125	0.125	32.9	1.25
**11**	-	-	1	-	32.9	1.25
**12**	0.333	0.667	-	-	32.9	1.25
**13**	-	0.333	-	0.667	32.9	1.25
**14**	-	-	-	1	65.8	2.50
**15**	0.333	-	0.667	-	32.9	1.25
**16**	0.333	0.333	-	0.333	32.9	1.25
**17**	-	0.667	-	0.333	32.9	1.25
**18**	-	0.333	0.667	-	16.4	0.625
**19**	0.625	0.125	0.125	0.125	32.9	1.25
**20**	0.333	0.333	0.333	-	32.9	1.25
**21**	1	-	-	-	65.8	2.50
**22**	0.125	0.125	0.125	0.625	32.9	1.25
**23**	-	-	0.667	0.333	32.9	1.25
**24**	-	1	-	-	32.9	1.25
**25**	0.667	-	0.333	-	65.8	2.50
**26**	0.333	0.333	0.333	-	32.9	1.25
**27**	0.25	0.25	0.25	0.25	32.9	1.25
**28**	-	-	1	-	32.9	1.25
**29**	0.667	-	-	0.333	65.8	2.50
**30**	0.667	0.333	-	-	32.9	1.25

SH = Summer savory essential oil (EO); OR = Oregano EO; CL = Cinnamon leaf EO; RT = Red thyme EO. “-” means no EO added.

**Table 4:** Decay index (DI) of tomatoes after 7, 14, 21 and 28 days of cold storage (4 ± 2 °C) (D7, D14, D21, and D28) and after the cold storage followed by 2 days of shelf life at 20 °C (D7+2, D14+2, D21+2, and D28+2), under control, active paper A and active paper B treatments.

Treatment	D7	D7+2	D14	D14+2	D21	D21+2	D28	D28+2
Control	1.0 ± 0.0	1.1 ± 0.1	1.1 ± 0.1	1.0 ± 0.0	1.0 ± 0.0	1.1 ± 0.2	1.3 ± 0.2	2.5 ± 0.2 a
Active paper A	1.0 ± 0.0	1.0 ± 0.0	1.1 ± 0.1	1.1 ± 0.1	1.1 ± 0.1	1.3 ± 0.3	1.2 ± 0.2	1.7 ± 0.5 b
Active paper B	1.0 ± 0.0	1.0 ± 0.0	1.0 ± 0.0	1.1 ± 0.2	1.1 ± 0.2	1.1 ± 0.1	1.1 ± 0.2	1.5 ± 0.1 b

Different letters for each evaluation time indicate significant differences among treatments according to Tukey’s test at *p* < 0.05.

**Table 5:** Effect of active papers on the sensory attributes of tomato fruits after 7, 14, and 21 days of cold storage at 4 ± 2 °C plus 2 days of shelf life at 20 °C evaluated through a consumer panel.

Treatment	Appearance	Firmness	Juiciness	Mealiness	Tomato Flavor	Off-Flavors	Global Acceptance
**D7+2**							
Control	7.3 a	7.9 a	7.6 a	3.9 a	6.8 a	2.9 b	7.3 a
Active paper A	7.9 a	7.1 a	7.3 a	4.3 a	6.5 a	4.9 a	6.3 a
Active paper B	7.6 a	7.2 a	7.2 a	4.6 a	6.4 a	4.7 a	6.5 a
**D14+2**							
Control	7.6 a	7.3 a	6.9 a	4.9 a	6.5 a	2.9 b	6.8 a
Active paper A	7.2 a	6.2 a	6.8 a	5.2 a	6.5 a	3.5 b	6.7 a
Active paper B	6.9 a	6.1 a	7.1 a	4.9 a	6.0 a	4.5 a	6.1 a
**D21+2**							
Control	7.2 a	6.1 a	6.6 a	4.6 a	5.9 a	2.6 b	7.0 a
Active paper A	6.6 a	6.2 a	7.2 a	3.8 a	6.4 a	4.5 a	6.8 a
Active paper B	6.5 a	5.9 a	7.0 a	4.5 a	5.9 a	4.2 a	6.7 a

Different letters in the same column for each of the storage periods indicate a significant difference (*p* < 0.05) between treatments according to the HSD Tukey multiple comparison test.

## Data Availability

The original contributions presented in this study are included in the article/Supplementary Material. Further inquiries can be directed to the corresponding author.

## Supplementary Materials

The following supporting information can be downloaded at: https://www.mdpi.com/article/10.3390/foods14162774/s1, Figure S1: Example of Minimal Inhibitory Concentration (MIC) determination in vapour phase. Antifungal activity results of *Botrytis cinerea grown* on Sabouraud Dextrose Agar (SDA) plates after incubation with different EO volumes: 0 µL-Control (a), 0.625 µL EO (b), 1.25 µL EO (c), 2.5 µL EO (d), 5 µL EO (e), and 10 µL EO (f). MIC corresponds to the minimal concentration of EO in which no fungal growth is found (in the example, MIC is determined at 65.8 µL EO added/L air, which corresponds to 2.5 µL EO added). Figure S2: Visual damage scale used for tomato, where (A) no damage (0% damage), (B) mild damage (10–15% damage), (C) moderate damage (25–50% damage), and (D) severe damage (>50% damage); Table S1: Composition of Oregano EO as analysed by direct injection GC-MS; Table S2: Composition of Summer savory EO as analysed by direct injection GC-MS; Table S3: Composition of Cinnamon leaf EO as analysed by direct injection GC-MS; Table S4: Composition of Red thyme EO as analysed by direct injection GC-MS; Table S5: Tomato fruit initial quality: total soluble solids (TSS) titratable acidity (TA), firmness (Durofel value) and colour values L*, a*, b*, and hue angle (h˚). The average and standard deviation is shown for each attribute.

## Abbreviations

The following abbreviations are used in this manuscript:

EO	Essential oil
RT	Red thyme essential oil
CL	Cinnamon leaf essential oil
OR	Oregano essential oil
SH	Summer savory essential oil
EO_mix_	Mixture of CL and OR (2:1 *v*/*v*)
MIC	Minimal Inhibitory Concentration
SDA	Sabouraud Dextrose Agar
DI	Decay Index

## References

[1] Food and Agriculture Organization of the United Nations (2013). Food Wastage Footprint: Impacts on Natural Resources.

[2] Food and Agriculture Organization of the United Nations (2014). Mitigation of Food Wastage Societal Costs and Benefits.

[3] European Commission Eurostat Statistics Explained—Food Waste and Food Waste Prevention—Estimates. https://ec.europa.eu/eurostat/statistics-explained/index.php?title=Food_waste_and_food_waste_prevention_-_estimates.

[4] Porat R., Lichter A., Terry L.A., Harker R., Buzby J. (2018). Postharvest Losses of Fruit and Vegetables during Retail and in Consumers’ Homes: Quantifications, Causes, and Means of Prevention. Postharvest Biol. Technol..

[5] Food and Agriculture Organization of the United Nations (2011). Global Food Losses and Food Waste—Extent, Causes and Prevention.

[6] Food and Agriculture Organization of the United Nations (2019). The State of Food and Agriculture. Moving Forward on Food Loss and Waste Reduction.

[7] Alegbeleye O., Odeyemi O.A., Strateva M., Stratev D. (2022). Microbial Spoilage of Vegetables, Fruits and Cereals. Appl. Food Res..

[8] Barth M., Zhuang H., Breidt F., Hankinson T.R., Sperber W.H., Doyle M.P. (2009). Microbiological Spoilage of Fruits and Vegetables. Compendium of the Microbiological Spoilage of Foods and Beverages.

[9] Pitt J.I., Hocking A.D. (2009). Fungi and Food Spoilage.

[10] Spadaro D., Droby S. (2016). Development of Biocontrol Products for Postharvest Diseases of Fruit: The Importance of Elucidating the Mechanisms of Action of Yeast Antagonists. Trends Food Sci. Technol..

[11] Milicevic D., Nesic K., Jaksic S. (2015). Mycotoxin Contamination of the Food Supply Chain—Implications for One Health Programme. Procedia Food Sci..

[12] Stoev S.D. (2015). Foodborne Mycotoxicoses, Risk Assessment and Underestimated Hazard of Masked Mycotoxins and Joint Mycotoxin Effects or Interaction. Environ. Toxicol. Pharmacol..

[13] World Health Organization Mycotoxins. https://www.who.int/news-room/fact-sheets/detail/mycotoxins.

[14] Punja Z.K., Rodriguez G., Tirajoh A., Formby S. (2016). Role of Fruit Surface Mycoflora, Wounding and Storage Conditions on Post-Harvest Disease Development on Greenhouse Tomatoes. Can. J. Plant Pathol..

[15] Hahn F. (2006). Rhizopus Stolonifer Detection by Sensing the Tomato Peduncle Scar. Biosyst. Eng..

[16] Doehlemann G., Ökmen B., Zhu W., Sharon A. (2017). Plant Pathogenic Fungi. Microbiol. Spectr..

[17] Charles M.T., Makhlouf J., Arul J. (2008). Physiological Basis of UV-C Induced Resistance to Botrytis Cinerea in Tomato Fruit. Postharvest Biol. Technol..

[18] Obande M.A., Tucker G.A., Shama G. (2011). Effect of Preharvest UV-C Treatment of Tomatoes (*Solanum lycopersicon* Mill.) on Ripening and Pathogen Resistance. Postharvest Biol. Technol..

[19] Stevens C., Liu J., Khan V.A., Lu J.Y., Kabwe M.K., Wilson C.L., Igwegbe E.C.K., Chalutz E., Droby S. (2004). The Effects of Low-Dose Ultraviolet Light-C Treatment on Polygalacturonase Activity, Delay Ripening and Rhizopus Soft Rot Development of Tomatoes. Crop Prot..

[20] Hua L., Yong C., Zhanquan Z., Boqiang L., Guozheng Q., Shiping T. (2018). Pathogenic Mechanisms and Control Strategies of *Botrytis cinerea* Causing Post-Harvest Decay in Fruits and Vegetables. Food Qual. Saf..

[21] Wu F., Lin Y., Zheng B., Wang H., Qu Z., Zhang X., Cai H., Li X., Feng S. (2024). 2-Phenylethanol Biocontrol Postharvest Tomato Gray Mold and Its Effect on Tomato Quality. Sci. Hortic..

[22] Elad Y., Williamson B., Tudzynski P., Delen N. (2007). Botrytis: Biology, Pathology and Control.

[23] Elad Y., Pertot I., Cotes Prado A.M., Stewart A., Fillinger S., Elad Y. (2016). Plant Hosts of *Botrytis* spp.. Botrytis—The Fungus, the Pathogen and Its Management in Agricultural Systems.

[24] Duda-Chodak A., Tarko T., Petka-Poniatowska K. (2023). Antimicrobial Compounds in Food Packaging. Int. J. Mol. Sci..

[25] Jahani M., Pira M., Aminifard M.H. (2020). Antifungal Effects of Essential Oils against *Aspergillus niger* in Vitro and in Vivo on Pomegranate (*Punica granatum*) Fruits. Sci. Hortic..

[26] Leyva Salas M., Mounier J., Valence F., Coton M., Thierry A., Coton E. (2017). Antifungal Microbial Agents for Food Biopreservation—A Review. Microorganisms.

[27] Reyes-Jurado F., Franco-Vega A., Ramírez-Corona N., Palou E., López-Malo A. (2015). Essential Oils: Antimicrobial Activities, Extraction Methods, and Their Modeling. Food Eng. Rev..

[28] Türkmenoğlu A., Özmen D. (2021). Allergenic Components, Biocides, and Analysis Techniques of Some Essential Oils Used in Food Products. J. Food Sci..

[29] Arraiza M.P., González-Coloma A., Andres M.F., Berrocal-Lobo M., Domínguez-Núñez J.A., Da Costa A.C., Navarro-Rocha J., Calderón-Guerrero C., El-Shemy H.A. (2018). Antifungal Effect of Essential Oils. Potential of Essential Oils.

[30] De Souza E.L., Lundgren G.A., De Oliveira K.Á.R., Berger L.R.R., Magnani M. (2019). An Analysis of the Published Literature on the Effects of Edible Coatings Formed by Polysaccharides and Essential Oils on Postharvest Microbial Control and Overall Quality of Fruit. Comp. Rev. Food Sci. Food Saf..

[31] Lucas-González R., Yilmaz B., Mousavi Khaneghah A., Hano C., Shariati M.A., Bangar S.P., Goksen G., Dhama K., Lorenzo J.M. (2023). Cinnamon: An Antimicrobial Ingredient for Active Packaging. Food Packag. Shelf Life.

[32] Manso S., Becerril R., Nerín C., Gómez-Lus R. (2015). Influence of pH and Temperature Variations on Vapor Phase Action of an Antifungal Food Packaging against Five Mold Strains. Food Control.

[33] Echegoyen Y., Nerín C. (2015). Performance of an Active Paper Based on Cinnamon Essential Oil in Mushrooms Quality. Food Chem..

[34] Stoleru E., Brebu M. (2021). Stabilization Techniques of Essential Oils by Incorporation into Biodegradable Polymeric Materials for Food Packaging. Molecules.

[35] Mendes J.F., Norcino L.B., Martins H.H.A., Manrich A., Otoni C.G., Carvalho E.E.N., Piccoli R.H., Oliveira J.E., Pinheiro A.C.M., Mattoso L.H.C. (2020). Correlating Emulsion Characteristics with the Properties of Active Starch Films Loaded with Lemongrass Essential Oil. Food Hydrocoll..

[36] Tomić A., Šovljanski O., Erceg T. (2023). Insight on Incorporation of Essential Oils as Antimicrobial Substances in Biopolymer-Based Active Packaging. Antibiotics.

[37] Turek C., Stintzing F.C. (2013). Stability of Essential Oils: A Review. Comp. Rev. Food Sci. Food Saf..

[38] Xue F., Gu Y., Wang Y., Li C., Adhikari B. (2019). Encapsulation of Essential Oil in Emulsion Based Edible Films Prepared by Soy Protein Isolate-Gum Acacia Conjugates. Food Hydrocoll..

[39] Yammine J., Chihib N.E., Gharsallaoui A., Ismail A., Karam L. (2024). Advances in Essential Oils Encapsulation: Development, Characterization and Release Mechanisms. Polym. Bull..

[40] Shao P., Yu J., Chen H., Gao H. (2021). Development of Microcapsule Bioactive Paper Loaded with Cinnamon Essential Oil to Improve the Quality of Edible Fungi. Food Packag. Shelf Life.

[41] Aguado R., Ferreira A.C.S., Gramacho S., Murtinho D., Valente A.J. (2022). Crosslinking of Surface-sizing Starch with Cyclodextrin Units Enhances the Performance of Paper as Essential Oil Carrier. Nord. Pulp Pap. Res. J..

[42] Oyom W., Zhang Z., Bi Y., Tahergorabi R. (2022). Application of Starch-based Coatings Incorporated with Antimicrobial Agents for Preservation of Fruits and Vegetables: A Review. Prog. Org. Coat..

[43] The European Parliament (2004). European Commission Regulation (EC) No 1935/2004 of the European Parliament and of the Council of 27 October 2004 on Materials and Articles Intended to Come into Contact with Food and Repealing Directives 80/590/EEC and 89/109/EEC.

[44] European Committee on Antimicrobial Susceptibility Testing (EUCAST) (2022). Method for the Determination of Broth Dilution Minimum Inhibitory Concentrations of Antifungal Agents for Conidia Forming Moulds.E.Def. 9.4.

[45] Tullio V., Nostro A., Mandras N., Dugo P., Banche G., Cannatelli M.A., Cuffini A.M., Alonzo V., Carlone N.A. (2007). Antifungal Activity of Essential Oils against Filamentous Fungi Determined by Broth Microdilution and Vapour Contact Methods. J. Appl. Microbiol..

[46] Badawy M.E.I., Rabea E.I. (2009). Potential of the Biopolymer Chitosan with Different Molecular Weights to Control Postharvest Gray Mold of Tomato Fruit. Postharvest Biol. Technol..

[47] Perdones A., Sánchez-González L., Chiralt A., Vargas M. (2012). Effect of Chitosan-Lemon Essential Oil Coatings on Storage-Keeping Quality of Strawberry. Postharvest Biol. Technol..

[48] (2017). Microbiology of the Food Chain—Preparation of Test Samples, Initial Suspension and Decimal Dilutions for Microbiological Examination—Part 1: General Rules for the Preparation of the Initial Suspension and Decimal Dilutions.

[49] (2013). Microbiology of the Food Chain.

[50] (2017). Microbiology of the Food Chain—Horizontal Method for the Detection and Enumeration of Enterobacteriaceae. Part 2: Colony-Count Technique.

[51] (2008). Microbiology of Food and Animal Feeding Stuffs—Horizontal Method for the Enumeration of Yeasts and Moulds—Part 1: Colony Count Technique in Products with Water Activity Greater than 0.95.

[52] (1994). Sensory Analysis—Identification and Selection of Descriptors for Establishing a Sensory Profile by a Multidimensional Approach.

[53] Bland M. (2000). An Introduction to Medical Statistics.

[54] Moussa H., El Omari B., Chefchaou H., Tanghort M., Mzabi A., Chami N., Remmal A. (2021). Action of Thymol, Carvacrol and Eugenol on *Penicillium* and *Geotrichum* Isolates Resistant to Commercial Fungicides and Causing Postharvest Citrus Decay. Can. J. Plant Pathol..

[55] Rao A., Zhang Y., Muend S., Rao R. (2010). Mechanism of Antifungal Activity of Terpenoid Phenols Resembles Calcium Stress and Inhibition of the TOR Pathway. Antimicrob. Agents Chemother..

[56] Xiang F., Zhao Q., Zhao K., Pei H., Tao F. (2020). The Efficacy of Composite Essential Oils against Aflatoxigenic Fungus *Aspergillus flavus* in Maize. Toxins.

[57] Abdolahi A., Hassani A., Ghuosta Y., Bernousi I., Meshkatalsadat M.H. (2010). In Vitro Efficacy of Four Plant Essential Oils against *Botrytis cinerea* Pers.:Fr. and *Mucor piriformis* A. Fischer. J. Essent. Oil Bear. Plants.

[58] Álvarez-García S., Moumni M., Romanazzi G. (2023). Antifungal Activity of Volatile Organic Compounds from Essential Oils against the Postharvest Pathogens *Botrytis cinerea*, *Monilinia fructicola*, *Monilinia fructigena*, and *Monilinia laxa*. Front. Plant Sci..

[59] Burt S. (2004). Essential Oils: Their Antibacterial Properties and Potential Applications in Foods—A Review. Int. J. Food Microbiol..

[60] Clemente I., Aznar M., Nerín C. (2019). Synergistic Properties of Mustard and Cinnamon Essential Oils for the Inactivation of Foodborne Moulds in Vitro and on Spanish Bread. Int. J. Food Microbiol..

[61] Nikkhah M., Hashemi M., Habibi Najafi M.B., Farhoosh R. (2017). Synergistic Effects of Some Essential Oils against Fungal Spoilage on Pear Fruit. Int. J. Food Microbiol..

[62] Lambert R.J., Skandamis P.N., Coote P.J., Nychas G.J. (2001). A Study of the Minimum Inhibitory Concentration and Mode of Action of Oregano Essential Oil, Thymol and Carvacrol. J. Appl. Microbiol..

[63] Sonboli A., Babakhani B., Mehrabian A.R. (2006). Antimicrobial Activity of Six Constituents of Essential Oil from Salvia. Z. Naturforschung C.

[64] Khanoonkon N., Rugthaworn P., Kongsin K., Sukyai P., Harnkarnsujarit N., Sothornvit R., Chollakup R., Sukatta U. (2022). Enhanced Antimicrobial Effectiveness of Synergistic Mixtures of Rambutan Peel Extract and Cinnamon Essential Oil on Food Spoilage Bacteria and Bio-based Food Packaging. J. Food Saf..

[65] El-Zehery H.R.A., Ashry N.M., Faiesal A.A., Attia M.S., Abdel-Maksoud M.A., El-Tayeb M.A., Aufy M., El-Dougdoug N.K. (2024). Antibacterial and Anticancer Potential of Bioactive Compounds and Secondary Metabolites of Endophytic Fungi Isolated from Anethum Graveolens. Front. Microbiol..

[66] Buendía−Moreno L., Sánchez−Martínez M.J., Antolinos V., Ros−Chumillas M., Navarro−Segura L., Soto−Jover S., Martínez−Hernández G.B., López−Gómez A. (2020). Active Cardboard Box with a Coating Including Essential Oils Entrapped within Cyclodextrins and/or Halloysite Nanotubes. A Case Study for Fresh Tomato Storage. Food Control.

[67] Buendía-Moreno L., Soto-Jover S., Ros-Chumillas M., Antolinos V., Navarro-Segura L., Sánchez-Martínez M.J., Martínez-Hernández G.B., López-Gómez A. (2019). Innovative Cardboard Active Packaging with a Coating Including Encapsulated Essential Oils to Extend Cherry Tomato Shelf Life. LWT.

[68] Campos A.D., Sena Neto A.R.D., Rodrigues V.B., Luchesi B.R., Moreira F.K.V., Correa A.C., Mattoso L.H.C., Marconcini J.M. (2017). Bionanocomposites Produced from Cassava Starch and Oil Palm Mesocarp Cellulose Nanowhiskers. Carbohydr. Polym..

[69] Buendía-Moreno L., Soto-Jover S., Ros-Chumillas M., Antolinos-López V., Navarro-Segura L., Sánchez-Martínez M.J., Martínez-Hernández G.B., López-Gómez A. (2020). An Innovative Active Cardboard Box for Bulk Packaging of Fresh Bell Pepper. Postharvest Biol. Technol..

[70] Becerril R., Nerín C., Silva F. (2020). Encapsulation Systems for Antimicrobial Food Packaging Components: An Update. Molecules.

[71] Chaidech P., Matan N. (2023). Cardamom Oil-Infused Paper Box: Enhancing Rambutan Fruit Post-Harvest Disease Control with Reusable Packaging. LWT.

