# Supplementary material for: Emulsion-Coated Active Papers Extend the Storage Life of Tomato Fruit
Source: Foods. 2025 Aug 9;14(16):2774. doi: 10.3390/foods14162774 (PMC12386085; doi:10.3390/foods14162774)
Supplement: Supplementary file 1 [file foods-14-02774-s001.zip › foods-3777777-supplementary.pdf]

SUPPLEMENTARY INFORMATION

Emulsion-Coated Active Papers Extend the Storage Life of Tomato Fruit

Laura Aguerri[1], Celia M. Cantín[2], Marinelly Quintero[1], Silvia Lóbez[1], Pedro Marco[3] and Filomena Silva[1,4,5]

[1]I3A – Aragon Institute of Engineering Research, University of Zaragoza, 50018 Zaragoza, Spain

[2]Estación Experimental Aula Dei (EEAD), CSIC, 50059 Zaragoza, Spain

[3]Centro de Investigación y Tecnología Agroalimentaria de Aragón (CITA), 50059 Zaragoza, Spain

[4]ARAID – Agencia Aragonesa para la Investigación y el Desarrollo, 50018 Zaragoza, Spain

[5]Faculty of Veterinary Medicine, University of Zaragoza, 50013 Zaragoza, Spain

Corresponding author: Filomena Silva

Phone: +34 876554222

E-mail address: filomena@unizar.es

Postal address: Departamento de Química, Facultad de Veterinaria, Calle de Miguel Servet, 177, 50013 Zaragoza, España

Table S1. Composition of Oregano EO as analysed by direct injection GC-MS.

Compound	Percentage
α/beta-Thujene	0.24
α-Pinene	0.31
Camphene	0.28
β-Pinene	1.26
3-Octanone	0.23
β-Myrcene	0.33
3-Octanol	0.12
Terpinolene	0.60
o-Cymene	9.98
D-Limonene	1.06
Eucalyptol	1.80
γ-Terpinene	4.04
Linalool	3.79
Camphor, (+)-	1.66
endo-Borneol	1.86
Terpinen-4-ol	0.97
α-Terpineol	0.38
α-Terpineol	0.99
Carvacrol methyl ether	0.55
Thymol	6.03
Carvacrol	56.23
Eugenol	0.47
Caryophyllene	2.86
Isocaryophyllene	0.10
Humulene	0.63
Caryophyllene oxide	2.85
Humulene oxide II	0.38

Table S2. Composition of Summer savory EO as analysed by direct injection GC-MS.

Compound	Percentage
α/beta-Thujene	0.76
α-Pinene	3.22
Camphene	1.33
β-Myrcene	2.47
Pseudolimonene	0.22
α-Terpinene	3.57
o-Cymene	15.04
D-Limonene	0.30
Eucalyptol	0.11
γ-Terpinene	21.86
Isolimonene	0.38
Linalool	0.39
endo-Borneol	0.28
Terpinen-4-ol	0.20
Thymol	3.61
Carvacrol	41.64
Caryophyllene	3.39
β-Bisabolene	0.60
Caryophyllene oxide	0.31
Unknown	0.32

Table S3. Composition of Cinnamon leaf EO as analysed by direct injection GC-MS.

Compound	Percentage
α-Pinene	1.41
Camphene	0.23
Benzaldehyde	0.18
β-Pinene	0.73
α-Phellandrene	1.20
α-Terpinene	0.11
o-Cymene	1.41
D-Limonene	0.88
Eucalyptol	0.21
Terpinolene	0.12
Linalool	2.81
Terpinen-4-ol	0.13
α-Terpineol	0.49
Chavicol	0.20
Cinnamaldehyde	2.24
Safrole	1.99
p-Thymol	0.12
Cinnamyl alcohol	0.14
Eugenol	61.48
Copaene	1.12
Caryophyllene	4.95
Acetic acid, cinnamyl ester	2.65
Humulene	1.01
Ledene	0.20
Eugenol acetate	5.22
Espatulenol	0.17
Caryophyllene oxide	1.02
Humulene oxide II	0.14
Cinnamaldehyde, 4-hydroxy-3-methoxy-	0.48
Benzyl Benzoate	6.82
Unknown	0.16

Table S4. Composition of Red thyme EO as analysed by direct injection GC-MS.

Compound	Percentage
α/beta-Thujene	0.94
α-Pinene	1.72
Camphene	0.60
β-Pinene	0.44
β-Myrcene	2.07
α-Phellandrene	0.26
3-Carene	0.12
α-Terpinene	2.47
o-Cymene	19.87
D-Limonene	0.95
γ-Terpinene	6.05
Terpinolene	0.44
Linalool	5.02
endo-Borneol	1.59
Terpinen-4-ol	1.57
Terpineol	0.21
Carvacrol methyl ether	0.45
p-Cymen-7-ol	0.60
Thymol	44.02
Carvacrol	6.27
Eugenol	0.25
Caryophyllene	2.04
Thymoquinol	0.57
Aromandendrene	0.17
Ledene	0.12
isoledene	0.18
Caryophyllene oxide	0.28
Unknown	0.72

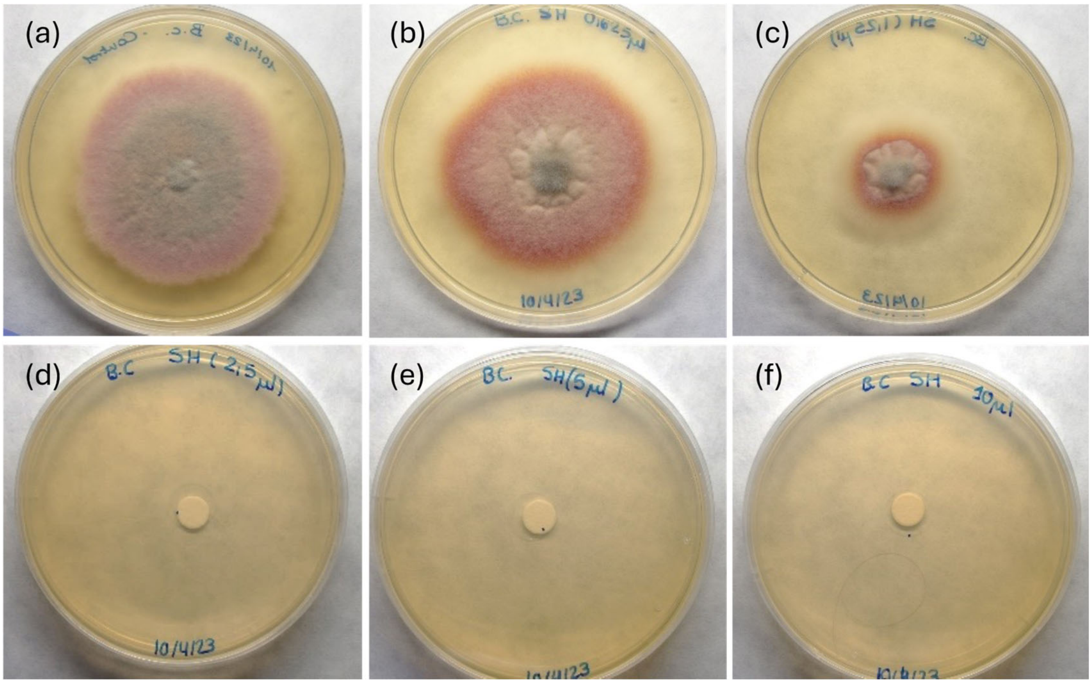

Figure S1. Example of Minimal Inhibitory Concentration (MIC) determination in vapour phase. Antifungal activity results of *Botrytis cinerea grown* on Sabouraud Dextrose Agar (SDA) plates after incubation with different EO volumes: 0 uL-Control (a), 0.625 µL EO (b), 1.25 µL EO (c), 2.5 µL EO (d), 5 µL EO (e) and 10 µL EO (f). MIC corresponds to the minimal concentration of EO in which no fungal growth is found (in the example, MIC is determined at 65.8 µL EO added/ L air, which corresponds to 2.5 µL EO added).

Table S5. Tomato fruit initial quality: total soluble solids (TSS) titratable acidity (TA), firmness (Durofel value) and colour values L*, a*, b* and hue angle (h˚). Average and standard deviation is shown for each attribute.

**TSS (˚Brix)**	**TA (%)**	**Firmness**	**L***	**a***	**b***	**h˚**
5.77 ± 0.4	6.85 ± 0.6	73.90 ± 6.6	37.52 ± 1.5	12.12 ± 2.7	20.23 ± 1.9	59.30 ± 5.4

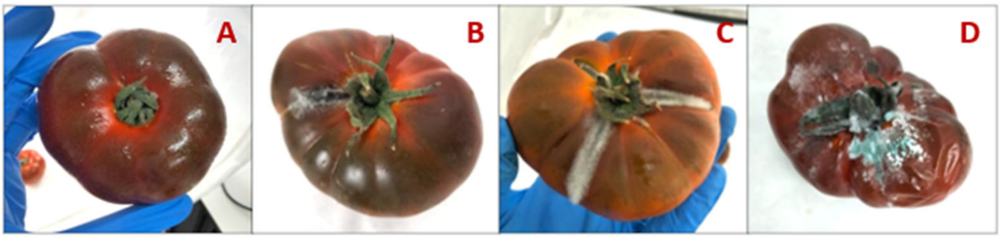

Figure S2. Visual damage scale used for tomato, where (A) no damage (0% damage), (B) mild damage (10–15% damage), (C) moderate damage (25–50% damage), and (D) severe damage (>50% damage).
